# Intraoperative Optical Coherence Tomography Imaging in Corneal Surgery: A Literature Review and Proposal of Novel Applications

**DOI:** 10.1155/2020/1497089

**Published:** 2020-09-11

**Authors:** Hiroshi Eguchi, Fumika Hotta, Shunji Kusaka, Yoshikazu Shimomura

**Affiliations:** ^1^Department of Ophthalmology, Kindai University, Faculty of Medicine, 377-2 Ohnohigashi, Osakasayama, Osaka 589-8511, Japan; ^2^Department of Ophthalmology, Fuchu Eye Center, 1-10-17 Hiko-cho, Izumi, Osaka 594-0076, Japan

## Abstract

Intraoperative optical coherence tomography (*i*OCT) is widely used in ophthalmic surgeries for cross-sectional imaging of ocular tissues. The greatest advantage of *i*OCT is its adjunct diagnostic efficacy, which facilitates to decision-making during surgery. Since the development of microscopic-integrated *i*OCT (MIOCT), it has been widely used mainly for vitreoretinal and anterior segment surgeries. In corneal transplantation, MIOCT allows surgeons to visualise structure underneath the turbid and distorted cornea, which are impossible to visualise with a usual microscope. Real-time visualisation of hard-to-see area reduces the operation time and leads to favorable surgical outcomes. The use of MIOCT is advantageous for a variety of corneal surgical procedures. Here, we have reviewed articles focusing on the utility of *i*OCT  and MIOCT in penetrating keratoplasty, deep anterior lamellar keratoplasty, Descemet stripping automated endothelial keratoplasty, and Descemet membrane endothelial keratoplasty. The applications of MIOCT to corneal surgery in terms of surgical education for trainees, emergency surgery, and novel surgery are also discussed, with our cases performed using RESCAN^®^ 700.

## 1. Introduction

Intraoperative optical coherence tomography (*i*OCT) is an imaging modality capable of showing real-time OCT images of the ocular tissue. This system confers advantages for both surgeon and the medical staff in the operating theatre during surgery. Although *i*OCT is now widely adopted to many ophthalmic surgeries for intraoperative cross-sectional imaging of the ocular tissues, there were some hurdles which conventional OCT modality must overcome before it is applied in the operating theatre [1]. The first OCT machines were desktop, stationary, and expensive, since they were initially designed for seated patients in outpatient clinic. Thus, relocating them to the operation theatre for intraoperative use was not practical. Thereafter, lightweight handheld OCTs were introduced, making it possible to bring the OCT machine into the operation theatre for patients in supine position [2–4]. However, handheld OCTs have limited use in the operation theatre since surgeons need to discontinue surgical manoeuvres when they obtain OCT images or require another medical staff for obtaining the image using this device, which translates to the OCT images not being truly “real time.” Although no article has reported the occurrence of intraoperative infections caused by handheld OCT, its use may increase the risk of intraoperative infection since it entails bringing nonsterile machine from outside of the operation theatre. Involuntary hand movement while using the handheld device also causes artifacts, which leads to lower quality of the acquired images [1]. Subsequently, Ray et al. [5] created their own mount for attaching a handheld OCT to the microscope, which allowed the surgeon or assistant to move the device above the patient's eye using the microscope foot pedal to ensure maintenance of sterility, improve image quality and reproducibility, and reduce image capture time. Similarly, Ehlers et al. [6, 7] fastened a handheld probe to the surgical microscope to provide increased stability of the probe and successfully obtained high quality *i*OCT images during vitreoretinal surgery.

Ehlers et al. were the first to demonstrate a microscope-integrated *i*OCT research system, which utilised a spectral domain OCT device attached in the space between the surgeon's eyepiece and microscope objective in a commercial surgical microscope [7, 8]. In recent years, OCT probes have been integrated into the microscope as commercially available products to enable true “real time” imaging of ocular tissues during the surgery, which was termed microscopic-integrated *i*OCT (MIOCT) [9]. The greatest advantage of *i*OCT is its adjunct diagnostic efficacy, which facilitates decision-making during surgery [6, 9, 10]. Its utility has been further enhanced with the advent of MIOCT, which allows the capture of cross-sectional images both on the microscope barrel and head-up monitor [10] without the need to discontinue surgical manoeuvre.

*i*OCT was initially developed for anterior segment surgery [11]. Thereafter, it has been applied to vitreoretinal surgeries, with numerous articles on such applications being published. These include its use for macular hole [5, 12, 13], epiretinal membrane [5, 14–16], retinal detachment [6, 17–19], and vitreomacular traction [15, 20, 21], among others [22–27]. Subsequently, its application has been expanded to include glaucoma surgery [28–31] and corneal transplantation [7, 9, 32–55]. To our knowledge, three systems are currently commercially available in worldwide: Rescan^®^ 700 (Carl Zeiss Meditec, Germany), OPMedT (OPMedT, Germany), and Bioptigen/Leica EnFocus (Leica, Germany). In this review, we will focus on the utility of *i*OCT or MIOCT for corneal surgeries, specifically penetrating keratoplasty (PK), deep anterior lamellar keratoplasty (DALK), Descemet stripping automated endothelial keratoplasty (DSAEK), and Descemet membrane endothelial keratoplasty (DMEK). New applications of MIOCT to both corneal surgery and in surgical education by introducing treated cases using Rescan^®^ 700 will be discussed. A report on the application of MIOCT to the latest corneal surgery will also be introduced.

## 2. Penetrating Keratoplasty (PK)

In PK, structures on the underside of the cornea, which are distorted at the host-graft interface, are hard to identify. If the structure in the anterior chamber underneath a severe peripheral corneal scar has changed during surgery, it is also difficult to detect the alteration using a typical microscope. During corneal suturing, after trephination of the host cornea, iris incarceration and iridocorneal adhesion can occur at anytime because eyeball is opened. MIOCT is useful in all the aforementioned situations, since it enables the visualisation of the endothelium layer, which runs beneath the host–graft interface [39]. The host-graft interface can be continually assessed during surgery by *i*OCT or MIOCT, which can help to prevent overriding/underriding of the graft and ensure proper apposition at the host-graft interface [40].

For the same reason as mentioned above, there could be value in the use of MIOCT in PK for educational purpose, especially for the verification of needle depth during suturing. Ideally, when suturing the graft to the host cornea, these structures' representative Descemet membranes (DMs) should be at the same height. If they were sutured at the different height, the grafted cornea may dissociate when the stitches are removed in the future. Therefore, the needle should be passed through a relatively deep corneal stroma, keeping the DMs of both host and graft cornea in mind. However, if the cornea is cloudy, it is not possible to determine the depth at where the needle is located using a typical microscope. If the host and graft were lifted with forceps so that these cross-sections could be visualised, the depth of the needle penetration into the cornea can be determined. However, such manoeuvre is impossible and undesirable in many cases. Therefore, the depth of the passed needle is usually estimated using the surgeon's hand.

**Figure 1:**
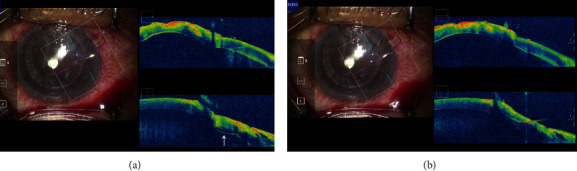
MIOCT images of the graft suture. (a) Needle penetration. When the 10-0 nylon needle was threaded through the graft, needle penetration was suspected based on the surgeon's judgment using his/her hand. MIOCT clearly showed that the needle penetrated and was located under the corneal endothelium (arrow). (b) MIOCT image after the corneal resuture. Once the needle is removed and the graft is restitched, the needle is threaded deep into the cornea appropriately.

Two studies have reported visualisation of the penetration depth of the syringe needle by *i*OCT in human [33] and porcine cornea [41], but no reports of *i*OCT confirmation of suture needle depth in the human corneal suturing in PK has yet been published. In the PK case presented in this study, confirmation of the position of the needle passing through the cornea was possible through the use of MIOCT. If the needle depth was found to be shallow, determining whether the thread should be rethreaded was made by the use of MIOCT and determining if the needle has unintentionally penetrated through the host or graft cornea (Figure 1(a)). The needle is then rethreaded accordingly, and the host and the graft are adjusted to the appropriate DM height (Figure 1(b)). Even for a skilled corneal surgeon, passing the suture needle into the cornea at the appropriate depth each time is not easy. Therefore, MIOCT would be useful in training of novice doctors for corneal suturing, especially in terms of verifying needle depth during the procedure. This verification may also be useful in emergency corneal suturing in cases of corneal rupture and corneal perforation.

## 3. Deep Anterior Lamellar Keratoplasty (DALK)

**Figure 2:**
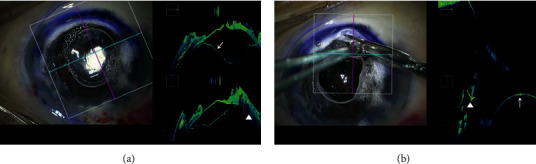
MIOCT images of DALK case. (a) Descemet membrane detachment. MIOCT shows cross-sectional images of both the Descemet membrane (arrow) detachment and the lamella dissected by air (arrow head). (b) Removal of the residual corneal stroma with scissors. The location between the Descemet membrane (arrow) and the scissors (arrow head) is roughly displayed by MIOCT, which contributes to the prevention of Descemet membrane rupture by preventing unintentional contact of the tip of scissors with the membrane.

In DALK, surgeons always need to assess the thickness of residual corneal stroma carefully during stromal excision. Even though it may appear that a significant amount of cornea has been removed when viewed from above under a typical microscope, MIOCT often reveals that more cornea remains than expected when the cross section is examined by MIOCT. Ehlers et al. reported in two articles that *i*OCT facilitated changes in dissection depth in 38–56% of cases [7, 9]. The use of air or ophthalmic viscoelastic bubbles during stromal excision [56] has led to the cornea becoming cloudy and the area underneath becoming completely invisible by a typical microscope. The surgeon therefore recognises DM detachment by observing big bubble formed in the corneal stroma which pushes the injected air in the anterior chamber. Although easy for a skilled surgeon, determining this using a typical microscope may be difficult for a novice DALK surgeon. Even in such circumstances, MIOCT can provide clear cross-sectional images of the stromal lamella, the bubbles in the stroma, and the movement of the DM (Figure 2(a)). When the DM has completely detached and the corneal stroma remaining over it is excised with scissors, full awareness of the depth of the DM should be kept in mind. If scissors were carelessly inserted into the deep stroma, the DM would rupture. In such situation, MIOCT can pinpoint a location between the DM and scissors (Figure 2(b)). MIOCT has also been reported as useful for assessing the location of the DM, for facilitating manual stromal excision, for assisting with the visualisation of the injected syringe needle into the stroma, and the assessing bare DMs [34, 35]. Furthermore, the measurement of the dissection depth of the corneal stroma by MIOCT has been reported to be an important factor of DALK success rate without conversion to PK [36]. These articles also substantiate the view that MIOCT is useful for education for novice DALK surgeons.

The utility of MIOCT for the visualisation of the misdirected air into the posterior chamber at the end of the DALK has been reported [39]. When microperforation of DM occurred during stromal excision, air injection into the anterior chamber should be performed. If the case had narrow angle, the air can be misdirected into the posterior chamber. In such case, MIOCT can detect the iris protrusion caused by air in the posterior chamber easily, which results in the prevention from high intraocular pressure in the early postoperative stage by injection fluid to let the air under the iris float immediately. It is often evident upon viewing the behaviour of the iris and air using a typical microscope, but the observation with MIOCT is more reliable for distorted cornea. MIOCT is useful for corneal surgeons in all proficiency levels in every surgical step of DALK.

## 4. Descemet Striping-Automated Endothelial Keratoplasty (DSAEK)

The advantage of MIOCT in DSAEK is its ability to visualise the relationship between the graft and the host cornea by viewing their cross-sections intraoperatively. This is true for both cases where the host cornea is relatively transparent and also in cases in which it is not. A study that used a handheld OCT noted that donor adherence can occur despite the residual interface space between the host cornea and the DSAEK graft at the end of the surgery, with the need for further research reported [42]. At this time, the space between the host cornea and the DSAEK graft might remain at the end of the surgery in many cases even if the surgeon had assumed that the graft had successfully adhered to the host cornea by air injection into the anterior chamber. The *i*COT has been suggested to be beneficial in elucidating the pathogenesis of phenomenon affecting surgical outcomes in DSAEK. Subsequently, another study which used a portable spectral domain OCT system with a customised microscope mount pointed out the association between the transient interface fluid, which can be observed intraoperatively on MICOT, and the texture interface opacity, which appears postoperatively, suggesting that intraoperative MIOCT findings are associated with postoperative outcomes [43].

**Figure 3:**
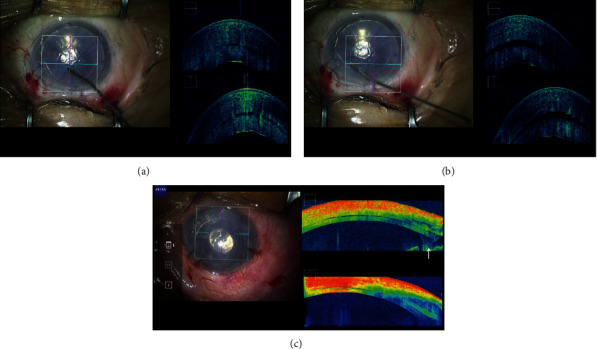
Residual interface space between the DSAEK graft and the host cornea. (a) Before corneal massage, a slight interface space between the DSAEK graft and the host cornea was observed. (b) Immediately after the corneal massage, the residual interface space widened. (c) DSAEK for advanced bullous keratopathy after multiple surgeries. Severe opacities in the cornea disturbed visualisation in the anterior chamber. MIOCT displayed a wide peripheral anterior synechia and a vitreous strand (arrow) touching the DSAEK graft.

After insertion of the DSAEK graft, the residual interface space between the graft and the host cornea is massaged on the host cornea to facilitate complete adhesion of the host and graft. However, the space widens after the massage in some cases, with the speculated cause being the inability of the curvature of the graft to match perfectly to that of the host cornea (Figures 3(a) and 3(b)). This is due to the cornea being not completely spherical and the DSAEK graft not always being punched in the centre of the grafted cornea each time. If the residual space between the host and the graft is widened after the massage, it would have been possible to attach the graft in all case by rotating the graft and performing an air injection only once, without the need for a repeat air or gas injection. Although further prospective studies are needed to warrant this procedure, this method was conceived only from the observations made by MIOCT.

Shazly et al. [49] and Pasricha et al. [50] reported that MIOCT is a valuable tool in performing DSAEK for severe opaque cornea cases in terms of viewing graft adherence to the host cornea. Similarly, the utility of MIOCT for determining the relationship between the DSAEK graft and the iris or vitreous in complicated case after multiple surgeries is also proposed. Patients who have undergone multiple internal ocular surgeries often have abnormal anatomical structures in their anterior chamber and corneal opacities. Severe opacities in the peripheral cornea interfere with the determination of the relationship between the edge of the DSAEK graft and the iris. In our patient with DSAKE who had advanced bullous keratopathy after multiple surgeries for cataract, retinal detachment, and glaucoma, MIOCT visualised the peripheral anterior synechia (PAS). Indeed, the finding can be observed upon preoperative examination in an outpatient clinic by usual OCT. However, after certain surgical manoeuvres, the structures in the anterior chamber would change. In this case, in the middle of the surgery, the PAS was found to be wider than expected, and a vitreous strand incarcerating into the surgical wound was observed to be disturbing the DSAEK graft attach in the centre of the host cornea (Figure 3(c)). After goniosynechialysis and anterior vitrectomy, the graft was attached satisfactorily. This case substantiates the view that MIOCT can facilitate central placement of the DSAEK graft and decrease the risk of postoperative rejection and secondary glaucoma following DSAEK by allowing detection of abnormal structures in the anterior chamber during surgery.

## 5. Descemet Membrane Endothelial Keratoplasty (DMEK)

**Figure 4:**
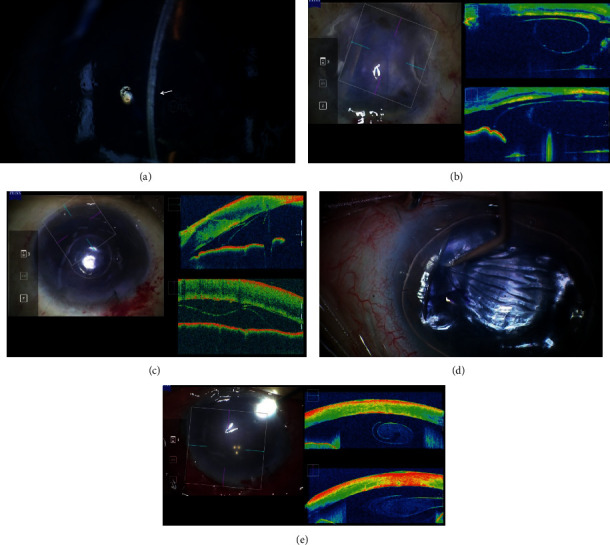
MIOCT images of DMEK cases. (a) Images of DMEK graft after insertion into the anterior chamber using a microscope-integrated slit-scan system. In the case of mild stromal oedema, the orientation of the DMEK graft can be ascertained (white arrow). (b) MIOCT images of the DMEK graft insertion into the anterior chamber. The graft orientation is clearly displayed as reverse even in the case of severe stromal oedema caused by Axenfeld–Rieger syndrome. In terms of images for decision-making, MIOCT images are much better than those of slit-scan systems. (c) Images after air injection for sticking the DMEK graft to the host cornea. The most peripheral part of the inserted DMEK graft in the anterior chamber is folded down. The MIOCT can display whether the graft folded toward the host or the anterior chamber. (d) Images of addressing the folded area by a scalpel. MIOCT image facilitates the manoeuvre of using the scalpel from the epithelial side. (e) MIOCT images of a DMEK graft, which never opened using any manoeuvre. The graft was curled strongly. MIOCT showed cloudiness inside the curled DMEK grafts, suggestive of viscous liquid which “glued” the graft on.

MICOT plays a major role in the success of DMEK. In every step in DMEK, MIOCT imaging facilitates decision-making, resulting in a high surgical success rate. Its most valuable utility is visualisation of DMEK graft orientation after its insertion into the anterior chamber. If stromal oedema was mild, the orientation of the DMEK graft could be ascertained with a microscope-integrated slit-scan system (Figure 4(a)). However, images captured by MICOT (Figure 4(b)) are superior. If the patient had a severe stromal oedema, MIOCT would be essential. If the initial surgery is unsuccessful and the DMEK graft is floating in the anterior chamber, the entire cornea would be marked oedema since the host endothelial cells have already been removed along with the DM. In the second surgery for graft correction in such cases, the orientation of the graft moving freely within the anterior chamber intraoperatively cannot be ascertained without MIOCT. Even if the DMEK graft was opened as its front and reversed successfully with air, its peripheral part may be folded down. If a scalpel is used to puncture the host corneal epithelium to correct the folded area, it is necessary to determine whether the endothelial cell side of the graft was folded in contact with the host cornea or to the anterior chamber. Making this decision would not be possible without MIOCT (Figures 4(c) and 4(d)).

Another application of MIOCT to DMEK would be its ability to aid in the decision to discontinue DMEK in the case where strong anterior chamber inflammation occurred during surgery. This study reports a case wherein a DMEK graft curled up, became fixed, and could not be opened with any subsequent manoeuvre. Preoperatively, the patient denied having any underlying disease, which may have cause intraocular inflammation. However, during surgery, the anterior chamber began to become rapidly cloudy after the iris was touched. Although there was no obvious fibrin aggregation, MICOT showed cloudiness inside the curled DMEK graft (Figure 4(e)). The rapid increase in anterior chamber inflammatory was concluded to have been due to blood-aqueous barrier break-down, causing the aqueous humor to become viscous and the curled DMEK graft to become impossible to open, as if it had been glued on. Eventually, DMEK was abandoned, but DSAEK was performed for correction later on. MIOCT greatly aids DMEK in all its stages after the graft insertion.

## 6. Emergency Surgery for Corneal Trauma

**Figure 5:**
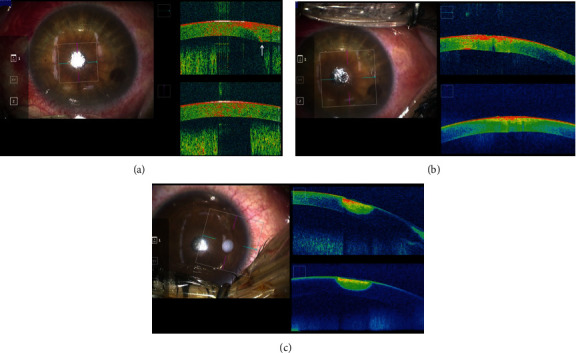
MIOCT images in cases of corneal trauma. (a) MIOCT image of a plant thorn piercing the deep cornea. Preoperative examination revealed that the DM near the plant thorn protruded (arrow), which was suggestive of corneal perforation. (b) MIOCT image immediately after removal of the plant thorn. No high-intensity shadow suggestive of residual foreign bodies in the cornea was found. (c) MIOCT image of a partial alkali burn in the cornea. The opacity was found to spread approximately to three-quarters of the corneal depth. If there is a delay between outpatient clinic examination and emergency surgery, the depth of the cornea opacity can be checked again to determine if the effect of alkali has progressed since the initial examination.

In this study, two cases of patients who underwent emergency surgeries for corneal trauma using MIOCT are reported. The first case made use of MIOCT for determining the depth of the foreign body in the cornea. A sharp and pointed plant thorn deeply pierced in the cornea and was removed in emergency surgery at the operation theatre. Preoperative examination by OCT showed the DM near the plant thorn protruded (Figure 5(a)). Removal at the outpatient clinic was deemed risky because the manoeuvre itself could penetrate the cornea. Otherwise, the cornea would have already perforated by plant thorn, and the anterior chamber would have collapsed after removal. A small amount of aqueous humor leaked after the removal as expected. MIOCT found no opacity in the wound (Figure 5(b)), which indicated that no foreign body was left in the corneal wound and the DM protrusion to have disappeared. Without MIOCT, corneal scraping would consider because the deep side of the wound cannot be found using a typical microscope. Performing scraping may cause both enlargement of the wound and more aqueous humor leakage, which results in the corneal scar.

The second case was a partial alkali burn of the cornea. In the case of a corneal alkali burn, the depth of the corneal opacity may have changed between the time of OCT imaging at the outpatient clinic and during the actual operation at the operation theatre, since alkali can melt protein. MIOCT can estimate the depth of corneal opacities in emergency surgery (Figure 5(c)). These two cases substantiate the view that MIOCT aids in decision-making during emergency surgery for corneal trauma.

## 7. Other Applications

Siebelmann et al. [57] reported the use of MIOCT for drainage of acute corneal hydrops in keratoconus. They performed the surgery using a combination of suturing and gas-aided reattachment of the DM, which may be facilitated by MIOCT. Tong et al. [58] reported the use of MIOCT in Bowman layer transplantation, which is a new type of corneal transplantation. MIOCT facilitates visualisation of the air-endothelial reflex dissection plane even under blood, oedema, or scarring. Schmidt et al. [59] reported the use of MIOCT in a corneal biopsy of a stromal opacity caused by immune deposits. They concluded that MIOCT assisted in identifying the corneal pathology for biopsy, which is in agreement with the findings in the aforementioned corneal trauma cases. Mazzotta and Caragiuli [60] reported the use of *i*OCT during corneal cross-linking and recommended intraoperative optical pachymetry evaluation before starting UV-A irradiation. Ghaffari et al. [61] and Pahuja et al. [62] also reported the use of *i*OCT or MIOCT during corneal cross-linking to evaluate the corneal pachymetry during the surgery. Kobayashi et al. [63] used MIOCT not for surgery but for evaluation of donor cornea tissues through the viewing chamber. They concluded that intact PK donors and prestripped DMEK donors are distinguishable by MIOCT, which may be beneficial for their institute where many corneal surgeons perform multiple corneal transplantations on the same day.

## 8. Current Limitations and Future Prospects

**Figure 6:**
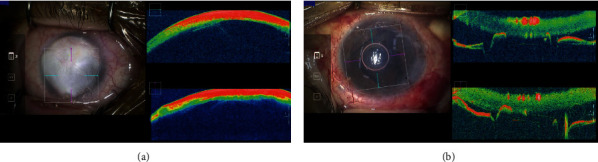
Limitations of MIOCT for corneal surgery. (a) MIOCT image of high-intensity congenital corneal opacity at the beginning of the surgery. Although the iris adhesions to the endothelial side of the cornea can be discerned, further detailed observation is not possible. (b) MIOCT image of DMEK case. An inverted ghost image of the cornea obscures the edge of the DMEK graft.

In cases of low-intensity corneal opacity, the structures in the anterior chamber can be ascertained. However, in cases of high-intensity corneal opacity, observation of the anterior chamber in detail is difficult (Figure 6(a)). The structures to be observed during anterior segment surgery are much thicker than the retina. Therefore, when observing the deep side of the anterior chamber, such as the iris or the angle, the cornea appears as an inverted ghost image superimposed on the structure to be observed (Figure 6(b)) [64]. This is a limitation of OCT that has not overcome since spectral-domain OCT for outpatient clinic use was made available. A small *i*OCT probe that is inserted into the eye has been developed, and its usefulness has been confirmed in animal experiments [65, 66]. It is hoped that in the future, this device will be able to be used like an intraocular endoscopes, helping to overcome the aforementioned limitations. In recent years, heads-up surgery has begun to gain popularity. A large MIOCT image displayed on a monitor while operating during heads-up surgery would amplify the benefits of MIOCT [67].

## 9. Conclusions

In conclusion, despite the aforementioned limitations, MIOCT aids the corneal surgeon in accurate and rapid intraoperative decision-making for all kinds of keratoplasty, thereby reducing operation times and improving postoperative outcomes for each procedure. MIOCT also has educational utility by allowing novice surgeons to be taught corneal suturing techniques and allows them to complete lamellar surgeries successfully. Novel applications of MIOCT have been reported, with more surgeons likely to use it in the future.

## Data Availability

Data sharing is not applicable to this article as no datasets were generated or analysed in the current study.
